# Plasma phylloquinone, menaquinone-4 and menaquinone-7 levels and coronary artery calcification

**DOI:** 10.1017/jns.2016.20

**Published:** 2016-12-29

**Authors:** S. Torii, Y. Ikari, K. Tanabe, T. Kakuta, M. Hatori, A. Shioi, T. Okano

**Affiliations:** 1 Department of Cardiovascular Medicine, Tokai University School of Medicine, Isehara, Japan; 2 Division of Cardiology, Mitsui Memorial Hospital, Tokyo, Japan; 3 Division of Nephrology, Endocrinology, and Metabolism, Tokai University School of Medicine, Isehara, Japan; 4 Department of Cardiology, Ibaraki Seinan Medical Center Hospital, Ibaraki, Japan; 5 Department of Cardiovascular Medicine, Osaka City University Graduate School of Medicine, Osaka, Japan; 6 Department of Hygienic Sciences, Kobe Pharmaceutical University, Kobe, Japan

**Keywords:** Vitamin K, Coronary artery calcification, Osteocalcin, Matrix Gla protein, BAP, bone-specific alkaline phosphatase, CAC, coronary artery calcification, MGP, matrix Gla protein, MK, menaquinone, NTX, N-terminal telopeptide, OC, osteocalcin, OPG, osteoprotegerin, PIVKA-2, protein induced by vitamin K absence of antagonist-2, PK, phylloquinone, PTH, parathyroid hormone, PWV, pulse wave velocity, t-ucMGP, total circulating uncarboxylated matrix Gla protein, ucOC, uncarboxylated osteocalcin

## Abstract

Vitamin K is considered to be involved in the pathological mechanisms of coronary artery calcification (CAC). Correlation between CAC and plasma vitamin K levels was studied. A total of 103 patients, with at least one coronary risk factor, were studied. CAC was measured using 64-slice multislice computed tomography (MSCT) and divided into three groups: none (CAC score = 0; *n* 25), mild to moderate (0 < CAC score < 400; *n* 52) and severe (CAC score > 400; *n* 26). Phylloquinone (PK) and menaquinone (MK)-4 and MK-7 were measured by HPLC-tandem MS. Mean age of patients was 64 (sd 13) years, of which 57 % were male. Median CAC score was 57·2. Median levels of PK, MK-4 and MK-7 were 1·33, 0 and 6·99 ng/ml, showing that MK-7 was the dominant vitamin K in this population. MK-7 showed a significant inverse correlation with uncarboxylated osteocalcin (ucOC, *P* = 0·014), protein induced by vitamin K absence of antagonist-2 (PIVKA-2, *P* = 0·013), intact parathyroid hormone (*P* = 0·007) and bone-specific alkaline phosphatase (*P* = 0·018). CAC showed an inverse correlation with total circulating uncarboxylated matrix Gla protein (t-ucMGP, *P* = 0·018) and Hb (*P* = 0·05), and a positive correlation with age (*P* < 0·001), creatinine, collagen type 1 cross-linked N-terminal telopeptide (NTX, *P* = 0·03), pulse wave velocity (*P* < 0·001) and osteoprotegerin (*P* < 0·001). However, CAC did not have a significant correlation with plasma levels of PK, MK-4 or MK-7. In conclusion, plasma MK-7, MK-4 or PK level did not show significant correlation with CAC despite the association between plasma vitamin K levels and vitamin K-dependent proteins such as ucOC or PIVKA-2.

Vitamin K is a cofactor for the enzyme responsible for the conversion of specific glutamyl residues to γ-carboxyglutamyl residues in several blood coagulation factors, bone-related proteins and other molecules. Vitamin K naturally exists in two forms, namely vitamin K_1_, also called phylloquinone (PK), and a group designated vitamin K_2_, also known as menaquinones (MK). PK is widely distributed in green and leafy vegetables, whereas MK exist preferentially in meats (MK-4), eggs (MK-4), curd (MK-8, MK-9), cheese (MK-8, MK-9) and fermented soyabeans (MK-7). PK is endogenously converted to MK-4^(^^1^^)^.

Arterial calcification is a pathological change found in atherosclerosis but the mechanisms involved have not yet been defined. In mice, gene knockout of the matrix Gla protein (MGP) caused death due to severe aortic calcification and aortic dissection^(^^2^^)^. In humans the level of inactive MGP correlated with the risk of CVD and mortality^(^^3^^–^^5^^)^.

The vitamin K antagonist, warfarin, induces arterial calcification in animals which can be reversed by the addition of MK^(^^6^^)^. A similar relationship between warfarin and arterial calcification has also been observed clinically^(^^7^^)^. Warfarin is a drug that blocks epoxide reductase, a recycling enzyme of vitamin K. Gene polymorphism of the epoxide reductase subunit correlated clinically with cardiovascular events such as stroke, myocardial infarction and aortic dissection^(^^8^^)^. A high dietary intake of PK as well as MK reduced coronary artery calcification (CAC)^(^^9^^,^^10^^)^ and prevented cardiovascular events in women^(^^11^^)^.

We performed an observational study to test the association between CAC and plasma levels of PK, MK-4 and MK-7.

## Methods

### Patient selection and study protocol

This study, the Vitamin K2 Super Minimizing Mineralization Trial (K2 SUMMIT-1), was performed at three hospitals (Tokai University Hospital, Mitsui Memorial Hospital and Ibaraki Seinan Medical Center Hospital), between 1 May 2008 and 28 December 2012. Patients with at least one coronary risk factor, e.g. hypertension, diabetes mellitus, hypercholesterolaemia, smoking, and a family history of coronary artery disease, were enrolled. Exclusion criteria were patients with an implanted coronary stent or pacemaker. The Institutional Review Board approved the study and all patients gave written informed consent. A medical history that included prior myocardial infarction, prior percutaneous coronary intervention, prior coronary artery bypass graft surgery, prior heart failure, prior stroke, or haemodialysis was obtained from each patient. The correlation between a CAC score and each coronary risk factor was studied.

### Cardiac multi-slice computed tomography data acquisition and analysis

A prospective non-enhanced coronary Ca scan was performed with a 64-slice MSCT scanner (Siemens) in all patients. For quantitative assessment of CAC, the Agatston score^(^^12^^)^ was calculated, using a 3 mm CT slice thickness and a detection threshold of 130 Hounsfield units (HU) involving ≧1 mm^2^ area/lesion (three pixels). A total CAC score was determined by summing individual lesion scores from each of four anatomical sites (left main trunk, left anterior descending artery, left circumflex artery, and right coronary artery)^(^^13^^)^. Patients were divided into three groups according to the clinical grading of CAC level: (1) none (Agatston score = 0); (2) mild to moderate CAC (0 < Agatston score < 400); and (3) severe CAC (Agatston score > 400).

### Measurements

Plasma was obtained from patients in the morning after overnight fasting and stored at −30°C. Vitamin K (PK, MK-4 and MK-7) was determined by HPLC–tandem MS with atmospheric pressure chemical ionisation (LC-APCI-MS/MS) as described previously^(^^14^^)^.

Total circulating uncarboxylated MGP (t-ucMGP) measurements were performed by Dr Cees Vermeer's group (Cardiovascular Research Center, Maastricht University, the Netherlands). Measurement methods and measurement errors have been reported previously^(^^15^^)^. Intact parathyroid hormone (PTH), osteocalcin (OC), uncarboxylated OC (ucOC), collagen type 1 cross-linked N-terminal telopeptide (NTX), bone-specific alkaline phosphatase (BAP), high sensitive C-reactive protein, intact PTH, oxidised LDL and protein induced by vitamin K absence of antagonist 2 (PIVKA-2) were measured by SRL Inc. Intra- and inter-assay CV were OC (3·4 %, 5·0 %), ucOC (1·3 %, 2·0 %), NTX (8·8 %, 11·9 %), BAP (2·5 %, 2·9 %), high sensitive C-reactive protein (0·9 %, 2·3 %), intact PTH (0·8 %, 3·0 %), oxidised LDL (6·2 %, 5·3 %), osteoprotegerin (OPG) (3·0 %, 5·0 %) and PIVKA-2 (5·7 %, 6·1 %).

Bone density was measured in the lumbar vertebra using a bone densitometer (DSC-900FX; Hitachi-Aloka Medical).

Ankle–brachial index and brachial–ankle pulse wave velocity (PWV) were measured using a vascular screening device (BP-203 RPE; Omron-Colin).

### Statistics

Continuous variables are presented as means and standard deviations in a normal distribution or as medians and interquartile ranges. Categorical variables are presented as absolute numbers and percentages. Univariate analysis was performed to assess the relationship between CAC and other parameters by calculating Spearman's rank correlation coefficients. To identify the primary predictor for CAC, ANOVA was performed. Statistical analysis was performed using SAS version 9.2 (SAS Institute, Inc.).

This study was registered as UMIN000002759.

## Results

Baseline patient characteristics are shown in Table 1. The mean age of the study population was 64 (sd 13) years, of which 57 % were male. Five patients (4·9 %) received haemodialysis. Pathology results for CAC, bone density, ankle–brachial index and PWV are shown in Table 2. The median value of the total Ca score was 57·2.

**Table 1.** Patient characteristics (Number of subjects and percentages; mean values and standard deviations)

	*n*	%
Patient number	103	
Age (years)		
Mean	64	
sd	13	
Male	59	57
Height (cm)		
Mean	160·1	
sd	9·0	
Body weight (kg)		
Mean	60·3	
sd	13·2	
BMI (kg/m^2^)		
Mean	23·3	
sd	3·9	
Hypertension	62	60
Hyperlipidaemia	70	68
Diabetes mellitus	40	39
Insulin-treated	4	4
Smoker	45	44
Prior myocardial infarction	9	9
Prior percutaneous coronary intervention	11	11
Prior coronary artery bypass graft surgery	3	3
Prior heart failure	12	12
Prior stroke	6	6
Haemodialysis patients	5	5
ACE-inhibitor or ARB	42	41
Antiplatelets	36	35
Statin	45	44

ACE, angiotensin converting enzyme; ARB, angiotensin receptor blocker.

**Table 2.** Baseline blood test, calcification score and pulse wave velocity (Mean values and standard deviations)

	Mean	sd
Hb (g/l)	136	17
Prothrombin time (international normalised ratio)	0·93	0·08
Albumin (g/l)	42·4	2·7
TAG ( mmol/l)	1·66	1·20
Alkaline phosphatase (IU/l)	231·1	69·9
Estimated GFR( ml/min per 1·73 m^2^)	60·4	25·7
Phosphate (mmol/l)	1·16	0·36
Ca (mmol/l)	2·28	0·23
ucOC (ng/ml)	5·8	11·9
OC (ng/ml)	8·9	16·0
ucOC:OC ratio	0·73	0·96
PIVKA-2 (mAU/ml)	23·9	11·1
Intact parathyroid hormone (pg/ml)	55·0	82·5
Bone-specific alkaline phosphatase (μg/l)	17·7	9·1
NTX (nmol BCE/l)	18·6	13·5
hsCRP (mg/l)	1320	3707
Osteoprotegerin (ng/ml)	101·4	60·5
Oxidised LDL (μmol/l)	3·00	1·35
t-ucMGP (nmol/l)	3315	1688
Phylloquinone (ng/ml)	1·6	1·3
Menaquinone-7 (ng/ml)	10·1	10·3
Menaquinone-4 (ng/ml)	2·1	15·9
Coronary artery Ca score (U)	591	1532
Bone density (g/cm^2^)	0·99	0·25
% Bone density	106·5	21·1
ABI	1·14	0·11
PWV (cm/s)	1682	395

IU, international units; GFR, glomerular filtration rate; ucOC, uncarboxylated osteocalcin; OC, osteocalcin; PIVKA-2, protein induced by vitamin K absence or antagonist- 2; mAU, milli absorbance units; NTX, N-terminal telopeptide; BCE, bone collagen equivalents; hsCRP, high sensitive C-reactive protein; t-ucMGP, total circulating uncarboxylated matrix Gla protein; ABI, ankle–brachial index; PWV, pulse wave velocity.

**Fig. 1.**
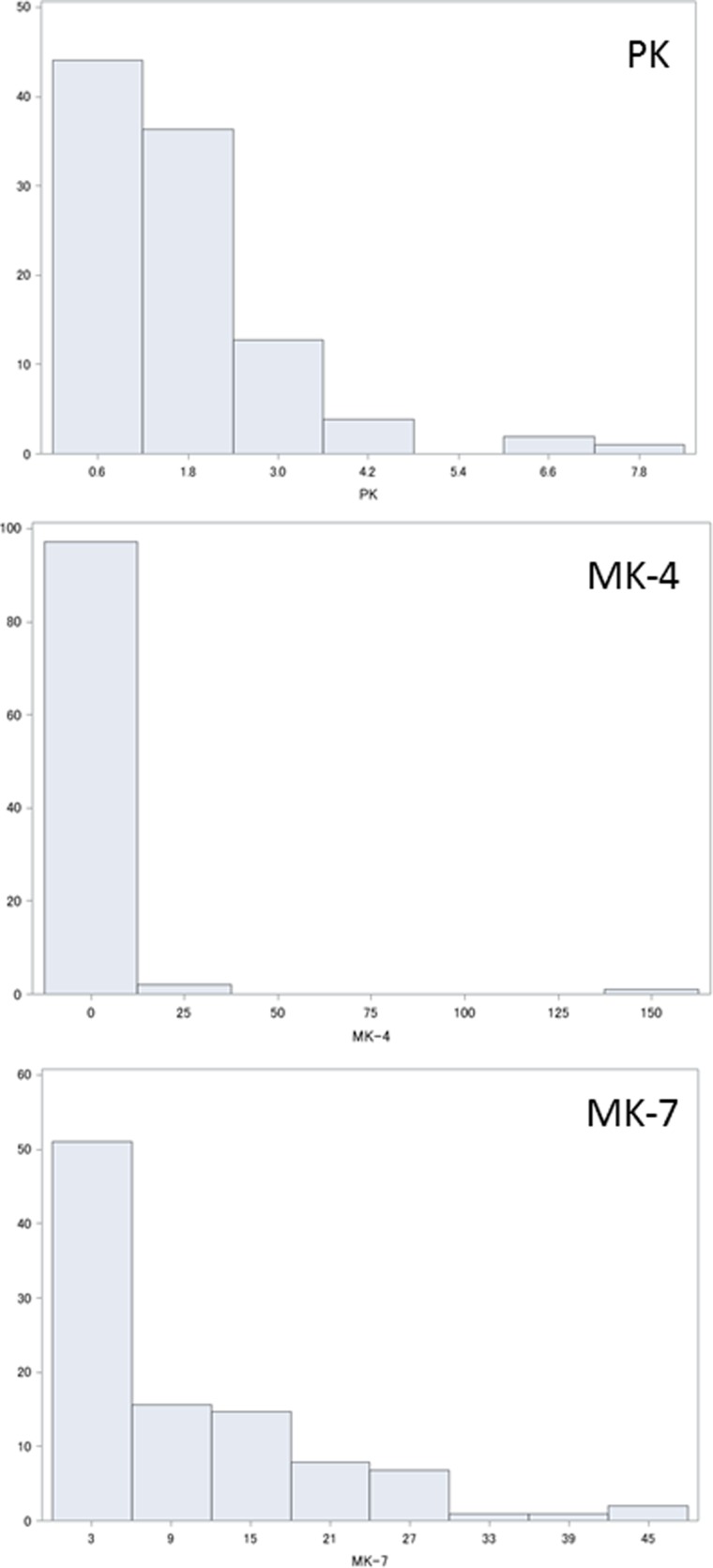
Histograms showing distribution (%) of plasma vitamin K levels (ng/ml): (a) phylloquinone (PK); (b) menaquinone (MK)-4; (c) MK-7.

Plasma levels of PK, MK-4 and MK-7 are shown in Fig. 1. Statistical analysis showed that PK, MK-4 and MK-7 were normally distributed as 1·64 (sd 1·26) ng/ml (median 1·33 ng/ml), 2·1 (sd 15·9) ng/ml (median 0, interquartile range 0–0·04, maximum 157·6 ng/ml) and 10·1 (sd10·3) ng/ml (median 6·99 ng/ml).

Associations between coronary calcification severity and risk factors are shown in Table 3. Patients with severe calcification were significantly older, and were significantly more likely to have a history of myocardial infarction, stroke or peripheral artery disease. Such patients showed significantly lower Hb levels and poor kidney function; they had significantly higher levels of NTX, OPG and ucOC, and a lower level of t-ucMGP. Patients with severe calcification also had significantly higher PWV values. However, plasma levels of PK, MK-4 or MK-7 did not correlate with the severity of CAC.

**Table 3.** Association between the severity of coronary calcification and clinical or blood test factors (Mean values and standard deviations, or percentages)

	No Ca (*n* 25): Ca score = 0		Mild to moderate Ca (*n* 52): 0 < Ca score < 400		Severe Ca (*n* 26): Ca score > 400		
	Mean	sd	Mean	sd	Mean	sd	*P*
Age (years)	56	14	65	11	71	12	<0·001
History of MI (%)	0		8		19		0·048
History of stroke (%)	0		4		15		0·05
Peripheral artery disease (%)	0		2		12		0·06
Use of aspirin (%)	12		31		46		0·03
Haemodialysis (%)	4		2		15		0·05
Hb (g/l)	141	18	137	14	129	19	0·03
BMI (kg/m^2^)	24·4	4·0	24·0	4·2	21·2	1·3	0·004
Albumin (g/l)	43	3	42	2	41	3	0·04
Creatinine (μmol/l)	124	230	80	88	239	398	0·03
Ca (mmol/l)	2·3	0·3	2·3	0·2	2·3	0·1	0·94
Phosphate (mmol/l)	1·2	0·5	1·1	0·3	1·2	0·3	0·94
ALP (IU/l)	258	78	213	49	242	88	0·02
Bone type ALP (μg/l)	16·1	7·8	17·2	7·2	20·3	12·9	0·23
Intact PTH (pg/ml)	52	58	41	21	85	148	0·09
Oxidised LDL (mmol/l)	3·2	1·6	3·1	1·4	2·7	0·9	0·32
NTX (nmol BCE/l)	16	6	17	11	25	11	0·03
OPG (ng/ml)	85	36	85	38	150	86	<0·001
t-ucMGP (nmol/l)	3455	1508	3659	1843	2529	1292	0·018
ucOC (ng/ml)	4·6	3·6	4·0	3·8	10·7	22·5	0·056
PIVKA-2 (mAU/ml)	21·6	5·3	23·8	8·1	26·1	18·1	0·36
PK (ng/ml)	1·4	1·0	1·8	1·3	1·6	1·3	0·46
MK-4 (ng/ml)	6·3	31·5	0·94	4·8	0·39	1·9	0·31
MK-7 (ng/ml)	8·7	9·1	10·5	10·1	10·8	11·1	0·72
hsCRP (mg/l)	2984	7169	918	1076	523	448	0·03
Bone matrix (g/cm^2^)	1·07	0·23	0·99	0·23	0·95	0·31	0·22
ABI	1·2	0·1	1·2	0·1	1·1	0·2	0·18
ba-PWV (cm/s)	1424	321	1655	281	2007	453	<0·001

MI, myocardial infarction; ALP, alkaline phosphatase; PTH, parathyroid hormone; BCE, bone collagen equivalents; NTX, N-terminal telopeptide; OPG, osteoprotegerin; t-ucMGP, total circulating uncarboxylated matrix Gla protein; ucOC, uncarboxylated osteocalcin; PIVKA-2, protein induced by vitamin K absence or antagonist-2; mAU, milli absorbance units; PK, phylloquinone; MK, menaquinone; hsCRP, high sensitive C-reactive protein; ABI, ankle–brachial index; ba-PWV, brachial–ankle pulse wave velocity.

MK-7, PK, TAG-adjusted MK-7 and TAG-adjusted PK data and related factors are listed in Table 4. MK-7 was inversely correlated with ucOC, PIVKA-2, creatinine, intact PTH, BAP and NTX, but positively correlated with percentage bone density in a statistically significant manner.

**Table 4.** Plasma levels of vitamin K and related factors (Mean values and standard deviations, or medians and ranges)

	Low		Medium		High		
	Mean	sd	Mean	sd	Mean	sd	*P*
MK-7 tertile group							
*n*	34		34		35		
MK-7 (ng/ml)							
Median	1·28		5·74		20·9		
Range	0–3·12		3·12–11·8		11·8–46·4		
Creatinine (μmol/l)	221	371	80	62	97	71	0·0241
% Bone density	103	21	103	17	113	23	0·0565
ucOC (ng/ml)	10·8	20·2	4·2	3·4	2·9	1·6	0·0149
PIVKA-2 (mAU/ml)	28·6	16·6	22·5	5·7	21·0	7·0	0·0133
Intact PTH (pg/ml)	89·3	135·5	46·6	15·6	29·9	21·5	0·0077
BAP (μg/l)	21·2	12·0	16·8	6·1	15·3	7·5	0·0188
NTX (nmol BCE/l)	23·4	21·6	16·6	5·3	15·8	5·6	0·0342
MK-7/TAG tertile group							
*n*	35		33		35		
MK-7/TAG (ng/ml per mmol)							
Median	0·011		0·045		0·161		
Range	0–0·031		0·031–0·078		0·082–0·584		
ucOC (ng/ml)	11·2	20·2	3·6	2·4	3·0	2·3	0·0080
PIVKA-2 (mAU/ml)	28·9	16·5	21·8	5·5	21·3	7·2	0·0077
Intact PTH (pg/ml)	89·4	132·6	42·8	21·2	32·1	22·3	0·0074
BAP (μg/l)	21·5	11·9	15·6	5·9	15·9	7·4	0·0089
PK tertile group							
*n*	34		34		35		
PK (ng/ml)							
Median	0·75		1·28		2·50		
Range	0·25–0·97		0·98–1·58		1·59–7·76		
Hb (g/l)	131	19	136	14	141	16	0·0249
TAG (mmol/l)	1·3	0·9	1·8	0·8	1·8	0·7	0·0094
BAP (μg/l)	22·2	11·6	15·5	7·1	15·6	6·3	0·0020
PK/TAG tertile group							
*n*	34		34		35		
PK/TAG (ng/ml per mmol)							
Median	0·0056		0·0099		0·0202		
Range	0·0018–0·0080		0·0083–0·0139		0·0141–0·0969		
Age (years)	61	13	61	15	70	8	0·0014
TAG (mmol/l)	2·4	1·6	1·5	0·7	1·1	0·6	<0·0001
BAP (μg/l)	21·1	11·2	15·2	6·4	16·8	8·2	0·0192
Oxidised LDL (mmol/l)	3·4	1·5	2·9	1·4	2·6	0·9	0·0320

MK, menaquinone; ucOC, uncarboxylated osteocalcin; PIVKA-2, protein induced by vitamin K absence or antagonist-2; mAU, milli absorbance units; PTH, parathyroid hormone; BCE, bone collagen equivalents; BAP, bone-specific alkaline phosphatase; NTX, N-terminal telopeptide; PK, phylloquinone.

## Discussion

In the studied population, MK-7 was the dominant vitamin K which had inverse correlation with ucOC, PIVKA-2, intact PTH and BAP. CAC showed an inverse correlation with t-ucMGP and Hb, and a positive correlation with age, creatinine, NTX, PWV and OPG. However, CAC did not have a significant correlation with plasma levels of PK, MK-4 or MK-7.

In this study, the medians of PK, MK-4 and MK-7 were found to be 1·33, 0 and 6·99 ng/ml, respectively, with levels of PK and MK-4 similar to those of previous reports^(^^16^^,^^17^^)^. On the other hand, the plasma MK-7 level was higher than those of previous reports: a median of 1·43 ng/ml was found by Fusaro *et al*. and a median of 3·92 ng/ml by Tsugawa *et al*.^(^^16^^,^^17^^)^. Such a discrepancy with the published literature may probably exist because this study was conducted in eastern Japan where fermented soyabeans known as natto, a food rich in MK-7, is popular. Tsugawa *et al*. reported that PK and MK-7, but not MK-4, correlated inversely with ucOC^(^^17^^)^. In comparison, we observed that MK-7, but not PK or MK-4, correlated inversely with ucOC and suggest this was because MK-7 may be the dominant vitamin K in this studied population. The population with the highest tertile of MK-7 exhibited a high percentage bone density, and low levels of ucOC, PIVKA-2, intact PTH, BAP and NTX. These data suggest that a high intake of MK-7 induced carboxylation to reduce ucOC and PIVKA-2, and to increase bone density with reduced bone destruction.

The group showing severe CAC were older in age, had a high PWV, and a frequent history of myocardial infarction, stroke and peripheral artery disease, indicating severe, whole-body atherosclerosis. As CAC is one of the known forms of atherosclerosis, such a relationship is conceivable^(^^18^^,^^19^^)^. Furthermore, the group with severe CAC also displayed chronic kidney disease and mineral and bone disorders, as suggested by high creatinine, low Hb and high NTX levels. Chronic kidney disease is also a well-known risk factor for CAC^(^^20^^,^^21^^)^ as also confirmed in this study.

CAC was also related to high levels of OPG, and low levels of t-ucMGP; these were also previously reported risk factors for arterial calcification^(^^2^^,^^3^^,^^22^^–^^24^^)^. The inverse association between t-ucMGP and calcification is generally explained by the tight binding of MGP's phosphoserine residues to hydroxyapatite, initially reported by Price *et al.*^(^^25^^)^. Despite the inverse correlation between MK-7 and ucOC, we remained puzzled as to the absence of a direct correlation between CAC and plasma vitamin K levels. The plasma vitamin K level reflects food intake over a short period of time but the ucOC level reflects a longer period of vitamin K insufficiency. Time-series studies in which the ucOC level was lowered by vitamin K supplementation showed that it remained unchanged at 15 d, but, by 30 d, reduction was observed^(^^26^^–^^28^^)^. Despite a weak association between CAC and ucOC (*P* = 0·056), CAC may correlate with the chronic status of vitamin K, but not with the plasma vitamin K level shown by a single measurement.

There are several limitations to this study. First, this study was conducted in eastern Japan where fermented soyabeans (natto) are a popular food, thus making the observed plasma MK-7 level higher than in previous studies. Second, as measurements of plasma vitamin K levels were not repeatedly performed, the possibility remains that errors were caused by the ingestion, by patients, of varying food content. Third, the number of patients studied was limited. If patient numbers were increased, a direct correlation between plasma vitamin K levels and CAC may be more readily observed. Finally, we did not measure dephospho-uncarboxylated MGP which is the most sensitive vitamin K marker presently available, and also a risk marker for CVD and mortality.

In conclusion, we could not show a direct correlation between plasma vitamin K levels and CAC. However, CAC correlated with markers of chronic insufficiency of vitamin K such as ucOC. This suggests that the chronic intake of sufficient vitamin K may have an inhibitory effect on CAC.
